# Common Belief and Make-Believe

**DOI:** 10.1007/s10670-025-00947-3

**Published:** 2025-05-08

**Authors:** Merel Semeijn

**Affiliations:** Rijksuniversiteit Groningen, Oude Boteringestraat 52, 9712 Groningen, The Netherlands

## Abstract

On Walton’s account of make-believe, unknown facts concerning the existence and nature of props can influence fictional truth. Inspired by Lewis’s and Walton’s discussions of import of fictional truth, I explore the shape and tenability of an alternative account that avoids such interference of unknown facts, by making fictional truth rely on participants’ *common beliefs* about props: conditional principles of generation are only valid if they quantify over props whose existence and nature is common belief between participants of the game of make-believe. I discuss two possible objections to the proposed account that are both based on the intuition that fictional truth should be something that is objective and independent of participants’ mental states.

## Walton’s Hidden Stump

In Walton’s (1990) highly influential theory of the representational arts from his seminal work *Mimesis as Make-Believe*, the activities that we undertake when we engage with the representational arts (e.g., paintings, plays, films, novels, sculptures, etc.) “are best seen as continuous with children’s games of make-believe. Indeed, [Walton] advocate[s] regarding these activities as games of make-believe themselves” (Walton, 1990, p.11). The activity that a child engages in when they play with a Barbie doll in the living room, is essentially the same kind of activity that their adult parent engages in when they go to the Museum of Modern Art to appreciate *The Starry Night*. Both child and adult use some object to help them imaginatively engage with a fictional world, containing its own set of fictional truths (separate from the actual world with its actual truths).

For Walton, the concept of imagination (or make-believe) plays a crucial role in the analysis of fiction and fictional truth (and hence in the analysis of the representational arts). In short, some proposition *p* is true in a game of make-believe *f* (i.e., is a fictional truth of *f*) iff participants of *f* are prescribed to imagine *p*,^1^ Different kinds of rules determine what we are to imagine in different games of make-believe. For instance, fictional truths can be generated by so-called ‘principles of (direct) generation’ (i.e., conditional rules), in combination with props (i.e., things in the actual world that principles of generation apply to). Let us consider Walton’s central example: Suppose Gregory and Eric are in a garden and decide to pretend that tree stumps (in the garden) are bears. Let us call this the ‘stump-game’. The stumps are the props that generate fictional truths in combination with conditional principle of generation *P*: 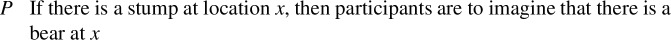 Assuming that there are no actual bears in the vicinity, the stump-game will also be guided by the following ‘inverse’ principle of generation $P^{i}$ (though this is usually left implicit in discussions of Walton): 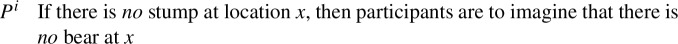


Thus, if there is a stump left of Gregory, the boys are to imagine that there is a bear left of Gregory, and hence it is fictional (true in the stump-game) that there is a bear there. Stumps can be props (as in this ad hoc stump-game), a Barbie doll can be a prop, but representational works of art such as paintings, plays, films and novels are also props, e.g., *The Hobbit* is a prop that (amongst other things) makes it true in my *Hobbit*-game that a hobbit finds a ring.

This paper investigates the interplay between props and prescriptions to imagine (and hence fictional truth). To this end I focus on the often noted (e.g., Lamarque (1991); Howell (1996); Toon (2010)) feature of Walton’s account that in a scenario where Gregory and Eric fail to spot a particular stump (e.g., because it is hidden behind a bush), it is *still* fictional that there is a bear there. Given their principle of generation and given the existence of the stump, it is true in their fiction that there is a bear lurking behind that bush (even though nobody imagined this).

An inactive hiding bear seems innocuous enough. However, I suggest that we can change the scenario such that the unknown fact of the hidden stump is detrimental to the ‘plot’ of the fiction: Suppose Gregory and Eric also agreed that it is true in their game that a group of ducklings is about to waddle by, and every bear in the garden has to be paralysed (e.g., by touching them) in order to ensure safe passage for the ducklings. Let us call this game the ‘ducklings-game’. The children paralyse every bear (i.e., touch every tree stump) they see, decide that they are done, congratulate each other, and terminate the pretence. Neither Gregory nor Eric ever finds out that there was an additional stump behind this bush. Unfortunately for the ducklings, on Walton’s account, it is still true in the ducklings-game that one bear is left awake. Gregory and Eric will never realise this, but they failed to rescue the ducklings in their fiction. Although the boys imagined being heroes and imagined ducklings happily waddling through the garden after they left, actually it is true in their game that the ducklings were slaughtered in a fury of yellow feathers, quacking and blood.

This paper explores the shape and possible complications (Sect. 4) of an alternative account of props and fictional truth (the ‘common belief account’) that avoids such unwelcome interference of unknown facts about props, by making fictional truth rely on participants’ ‘common beliefs’ about props. Before presenting the proposal (Sect. 3), I will present its main inspiration: Lewis’s (1978; 1983) and Walton’s (1990) use of the notion of common belief in their discussions of principles for import of fictional truth (Sect. 2).

## Imported Fictional Truth and Common Belief

The current paper proposes an alternative to Walton’s theory of how props generate fictional truth when combined with principles of direct generation. Before turning to this proposal, I will, in the current section, discuss a separate but parallel discussion, i.e., the discussion of principles for the ‘importation’ of fictional background information (or ‘principles of implication’ Walton (1990)). As we will see, the discussion on fictional truth by import displays important parallels to the discussion on fictional truth through props, and hence will function as a stepping stone for the presentation of this paper’s central proposal in Sect. 3 (see also Sect. 3.1.2).

### Imported Fictional Truth

It is a commonplace that there are more things that are true in a fiction than those that are explicitly stated or shown. For instance, it is an explicit, direct, or – in Walton’s,^2^ (1990) terminology – ‘primary’ fictional truth of the Sherlock Holmes novels that the rooms at 221B Baker Street “consisted of a couple of comfortable bed-rooms and a single large airy sitting-room” (Doyle, *A Study in Scarlet*). This is explicitly stated in the text of the Sherlock Holmes novels (i.e., the prop in the Holmes-game) and hence we are mandated to imagine this (i.e., it is true in the fiction). However, it is *also* true in the fictional world of the Holmes stories that water is $\hbox {H}_{2}$O, that whales are mammals, and “that Holmes does not have a third nostril” (Lewis, 1978, p.41), even though this is never stated in the novels, nor follows from anything that was stated in the novels. Rather, these fictional truths were imported. Import of fictional truth happens not just in literary fictions, but occurs across media. Consider a painting such as *La Grande Jatte*. As Walton (1990) writes:

It is fictional in *La Grande Jatte* that the couple strolling in the park eat and sleep and work and play; that they have friends and rivals, ambitions, satisfactions, and disappointments; that they live on a planet that spins on its axis and circles the sun, one with weather and seasons, mountains and oceans, peace and war, industry and agriculture, poverty and plenty; and so on and on and on. (p.142)

All of this is true in *La Grande Jatte* even though none of this is explicitly shown. The only things that we are shown (the primary fictional truths) are that there are people lounging and strolling in front of a lake.

### The Common Belief-Move

Several authors (e.g., Beardsley (1981); Lewis (1978); Ryan (1980); Wolterstorff (1980); Walton (1990)) have discussed principles of importation for such implicit, indirect or ‘implied’ (Walton, 1990) fictional truths. They often compare the intuitive appeal of two analyses: one that takes the *actual world* as a basis for fictional background information, and one that takes the audience’s *beliefs about the actual world* as a basis. The leading question in these discussions is whether unknown or little-known facts of nature should be allowed to influence fictional truth.

In the following, I focus on discussions on importation where the latter principle is formulated using the concept of *common* belief: Lewis’s (1978) discussion of his Analysis 1 and 2, and Walton’s (1990) discussion of his Reality Principle and Mutual Belief Principle. In his seminal paper ‘Truth in Fiction’, Lewis (1978) provides an analysis of the ‘In fiction *f*, $\phi$’-operator as an intensional operator, i.e., as quantifying over possible worlds. Lewis’s discussion focuses on truth in literary fictions, i.e., in written stories. Walton’s (1990) discussion in *Mimesis as Make-Believe* (chapter 4, section 3) closely follows Lewis, but broadens the scope by providing analyses for the import of fictional truth in any kind of medium (including literary fictions, but also, e.g., paintings, plays, films, etc.).

Lewis discusses several modal analyses for truth in fiction, of which Analysis 1 and 2 are the final (and, according to Lewis, best) candidates. Lewis’s Analysis 1 takes the actual world as basis for background information that is imported into the (literary) fiction. Roughly, a fictional world is as much like the actual world as the text allows. The fiction operator is thus analysed as a counterfactual, i.e., what is true in *f* is what would be true if *f* were told as known fact in our world. In other words, we take the actual world as our ‘starting point’ and see what it would be like if the things stated in *f* were told as known fact. Here is a simplified representation of Analysis 1 for fictional truth in literary fictions: 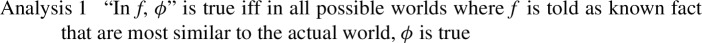 Walton (1990) provides a similar counterfactual analysis for truth in any kind of fiction (verbal, visual, etc.) called the Reality Principle (RP): 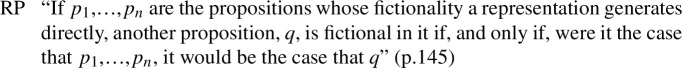 Walton also understands his counterfactual along Stalnaker-Lewis (Stalnaker, 1968; Lewis, 1973) lines, i.e., in terms of possible worlds that are more or less similar to the actual world. In other words, RP tells the fiction interpreter to ask themselves what the real world would be like if the propositions whose fictionality are generated directly, were in fact true. Hence, like Analysis 1, RP makes fictional worlds as much like the actual world as the relevant props allow.

Analysis 1 makes it true in the Sherlock Holmes novels that 221B Baker Street has one living room. Since this is explicitly stated in *f*, this is true in all worlds where *f* is told as known *fact*. For Walton, the fictional truth that 221B Baker Street has one living room is one that is (more or less, see especially (Walton, 1990, chapter 4, section 4) directly generated by the prop.

Moreover, both Analysis 1 and RP make it true in the Sherlock Holmes novels that water is $\hbox {H}_{2}$O (and that whales are mammals, etc).^3^ Consider Analysis 1: Worlds where the Sherlock Holmes novels are told as known fact *and* water is $\hbox {H}_{2}$O are more similar (or ‘closer’) to the actual world than worlds where the Sherlock Holmes novels are told as known fact and water is *not* $\hbox {H}_{2}$O. Now consider RP: Worlds where all primary truths of the Sherlock Holmes novels are true *and* water is $\hbox {H}_{2}$O are more similar to the actual world than worlds where all primary truths of the Sherlock Holmes novels are true and water is *not* $\hbox {H}_{2}$O.^4^ Analysis 1 and RP thus correctly predict the fictionality of such background information.

Analysis 1 and RP also make little-known and unknown facts relevant to fictional truth. On Analysis 1 and RP, whatever is actually the case will also be true in the fiction (unless explicitly contradicted by it). But, given that my partner went to the supermarket yesterday, is it then also true in the Sherlock Holmes novels that my partner went to the supermarket yesterday? As Walton writes, if we adopt RP “[i]t will be fictional in “Goldilocks and the Three Bears” that Tenzing and Hillary achieved the first ascent of Mount Everest and Neil Armstrong the first landing on the moon” (Walton, 1990, p.148). Must we accept this “clutter” (Walton, 1990, p.148) of fictional truths into fictions that are not in any way about these events? We may consider such import odd but relatively harmless (similar to the inactive hiding bear in the stump-game). Especially if we, as Walton suggests, recognise that we are not expected to focus on these fictional truths, but only on those highlighted by the story.

However, as both Lewis and Walton acknowledge, little-known or unknown facts that lurk in the background can turn out to be detrimental to the plot of a fictional narrative. This can lead to counter-intuitive results. Lewis gives us the example of the Holmes story *The Adventure of the Speckled Band*. In this story, Holmes claims to have solved a murder case by showing that someone has been killed by a viper that climbed up a bell rope. However, it is *not* explicitly stated in *The Adventure of the Speckled Band* that Holmes is correct. In other words, the text of the Sherlock Holmes stories does *not* state that the viper indeed climbed up a bell rope (only that Holmes said that it did). Famous zoologist Gans (1970) – working with (something like) Analysis 1 or RP – argued that, since vipers cannot actually climb ropes, either it is true in *The Adventure of the Speckled Band* that the snake reached its victim some other way, or Holmes has not solved the case at all. Either option is counter-intuitive: Holmes is always right! Similarly, Walton describes a hypothetical story, let us call it *The Edge of Reason*, invented by someone “in a culture in which it is universally and firmly agreed that the earth is flat and that to venture too far out to sea is to risk falling off” (Walton, 1990, p.150). In *The Edge of Reason*, a group of bold mariners sail very far out to sea. However, nowhere in *The Edge of Reason* is it stated that the earth is flat, or that sailing far out is dangerous. Applying Analysis 1 or RP would make it true in *The Edge of Reason* that in fact the earth is round and the mariners were never in any real danger. According to Walton, to do so would ruin a good adventure story because it is (presumably) necessary that it is true in *The Edge of Reason* that the earth has a dangerous edge, in order to establish the fiction’s dramatic effect.

To avoid these unwelcome consequences of Analysis 1, Lewis introduces Analysis 2. A simplified representation: 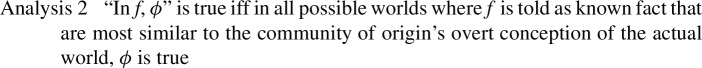 The “community of origin’s overt conception of the actual world” consists in the ‘general common beliefs’ about the actual world in the community of origin of the relevant fiction. *p* is ‘common belief’ (see e.g., Lewis (1969); Schiffer (1972)) within some community c iff everyone in c believes that *p*, everyone in c believes that everyone in c believes that *p*, everyone in *c* believes that everyone in *c* believes that everyone in *c* believes that *p*, etc.^5^ Likewise, Walton (1990) provides us with the Mutual Belief Principle (MBP) which is modelled after Lewis’s proposal: 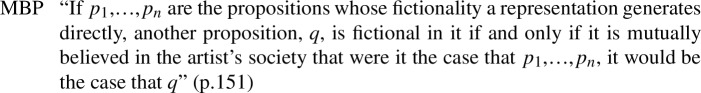 Here ‘mutual belief’ is just another term for general common belief.

Analysis 2 and MBP work very similar to respectively Analysis 1 and RP, except that not the actual world, but the relevant *community’s common conception of* the actual world is taken as a basis for our counterfactual reasoning. Analysis 2 and MBP still import truths such as that water is $\hbox {H}_{2}$O into the Sherlock Holmes fiction. This was common (or – in Walton’s terminology – mutual) belief between Doyle and his readers. However, information such as that my partner went to the supermarket yesterday or that vipers cannot climb ropes is *not* imported into the fiction. Although a single reader of the Sherlock Holmes novels may personally have believed either of these things, (and it may even become overt belief some time after the fiction has been published) it was *not* common belief within the community of origin (or – in Walton’s terminology – the artist’s society) and hence *not* part of what is true in the Sherlock Holmes novels. Similarly, the information that the earth is round is not imported into *The Edge of Reason*. In the community of origin of *The Edge of Reason* it was common belief that the earth is flat, and hence it will be true in *The Edge of Reason* that the earth is flat, and hence that the mariners were in real danger.

Both Lewis and Walton discuss various areas where Analysis 1, 2, RP and MBP fall short of a complete account of fictional truth when measured against actual practice (for a recent overview of criticism of Lewis’s analysis of fictional truth see García-Carpintero (2022)).^6^ The details of these discussions will not matter to us now. Moving forward, what is relevant to the discussion of the current paper is the ‘common belief-move’ that both authors make in their discussion of implicit fictional truths, and what prompted them to make it, i.e., the unwelcome interference of unknown matters of fact in the generation of fictional truth.

## Common Beliefs About Props

In this section I explore to what extent a similar common belief-move would be possible in Walton’s framework to deal with unknown facts *about props* influencing fictional truth. In other words, I will explore whether the notion of common belief may also be relevant for the analysis of *explicit* fictional truths (rather than just for the analysis of *implicit* fictional truths as discussed in Sect. 2).

### Motivation

At this point the reader may wonder about the motivation for such a project, especially since (as will become clear in Sect. 3.2), it leads to a notion of fictional truth that departs from Walton’s quite a bit.

#### Mixed Intuitions

The main motivation behind this project is the observation that intuitions concerning fictional truth in the ducklings-game scenario are unclear and not uniform across people. Whether the reader thinks that there is a problem to solve here (or whether they think that the current paper proposes an unnecessary twist on an established theory), is going to depend on their intuitions concerning fictional truth in the ducklings-game scenario. Although some theorists report experiencing no unease with Walton’s prediction of the ducklings’ death, many of my interlocutors (theorists and laypeople) report some level of uncertainty concerning fictional truth. It is not obvious to this latter group that we should indeed accept that (without Gregory or Eric ever knowing this) the children failed to rescue the ducklings in their fiction. There seems to be at least *some *intuitive appeal to giving a little more authority over fictional truth to Eric and Gregory, given that it is *their* game. The current paper explores to what extent we can make sense of this conflict in intuitions. Given that it is at least not obvious that Walton’s theory offers the right predictions in the ducklings-game scenario, we thus have a theoretical justification for exploring alternative analyses of fictional truth that may account for this.

Some have responded to this way of justifying the project of the current paper by questioning the described intuitions through further exploration of the ducklings scenario. Suppose, for instance, that Eric and Gregory *do* end up finding the hidden stump the next day. We might expect them to respond by saying (1) or (2): 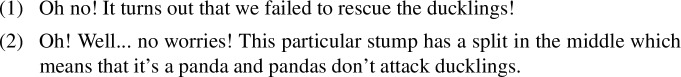 We could take the legitimacy of a response such as (5) as *prima facie* evidence that (at least the children themselves accept that) a Waltonian analysis of the game that Gregory and Eric played the day before is indeed right. We could take the legitimacy of a response such as (6) as *prima facie* evidence that, even if we *do* assume a Waltonian analysis of fictional truth, the children will still find a way to avoid the ducklings’ death.

I see no problem in agreeing that these are possible (or, indeed, likely) responses by Eric and Gregory in case they find the hidden stump the next day. However, when testing our intuitions concerning fictional truth, it is important to keep the ducklings-scenario ‘clean’ by assuming that the children in fact do *not* find that hidden stump after playing their game. Otherwise (given that it is generally difficult to separate talk and thoughts *about* a pretence from talk and thoughts *within* a pretence, see e.g., Recanati (2018)), we risk turning the ducklings-scenario into one where the game/pretence *continues* the next day. It is no wonder that in cases of continued pretence in which the children find a stump *after* the ‘slaughter-deadline’, the children indeed accept and imagine that the ducklings have died (or attempt to change the rules of their game to prevent this as in (6)). As will become clear shortly (Sect. 3.2), Walton’s original theory and the alternative common belief account proposed in this paper actually make the same predictions concerning fictional truth in these scenarios. Simply put, if the game continues thus the next day, Gregory and Eric will come to commonly believe (while still playing the game) that there is a stump (that they missed earlier) at the relevant location. This will also make it fictionally true that the ducklings died on the common belief account.

In sum, in case the reader wishes to make up their own mind, I suggest they gauge their intuitions for a ‘clean’ scenario in which the ducklings-game terminates *definitely* before the slaughter-deadline, and Gregory and Eric do *not* end up finding the hidden stump at a later time, i.e., consider games where it is unquestionably the case that there is a final verdict on what is true in the fiction that (at least on Walton’s account) deviates from what the children imagined.

#### Theoretical Parallel

Second, we can motivate the current project by drawing a parallel between the discussions on import and the discussion of the ducklings-game. The ducklings-game scenario mirrors Lewis’s bell rope scenario and Walton’s *The Edge of Reason*; these scenarios all raise the question of whether unknown facts of nature should be allowed to influence fictional truth (implicit fictional truth in the bell rope- and *The Edge of Reason*-cases, explicit fictional truth in the ducklings-game). In all three scenarios the unknown fact has far-reaching consequences for the plot of the fiction.

Just as for the ducklings-game scenario, intuitions vary concerning the correct analysis of imported fictional truth. Whereas Lewis remains neutral with respect to whether Analysis 1 or 2 is to be preferred, Walton suspects that MBP ‘wins’ over RP whenever they make conflicting predictions (Walton, 1990, p.156-157).^7^ Possible exemptions to this rule are predictions made about evaluative claims being true in a fiction, i.e., Walton suggests that RP may fare better with respect to moral facts (cf. the debate on ‘imaginative resistance’ Gendler (2000); Weatherson (2004)). So, at least in some cases, it is not obvious that MBP is superior to RP. Moreover, intuitions concerning fictional truth in cases like the bell rope scenario and *The Edge of Reason* probably also vary across people. It is not clear to everyone (e.g., it wasn’t to Gans (1970)) that MBP or Analysis 2 indeed gives the correct predictions in these cases.

Drawing this parallel gives us a meta-theoretical motivation to explore whether (just as Lewis and Walton resort to the notion of common belief in their discussion of import) a similar common belief-move would be possible for the ducklings-game. If theorists (including Walton) question whether we should allow unknown facts of nature to ruin the plot of a fiction in the case of *implicit * fictional truths, why would we (without questioning) let unknown facts of nature ruin the plot of a fiction in the case of *explicit* fictional truths? The current paper investigates this previously unexplored part of the logical space of possible analyses of fictional truth, brought to light by the ducklings-game scenario.

Note that, although the current proposal (concerning the analysis of explicit fictional truths) is thus inspired by Walton’s (and Lewis’s) discussion of implicit fictional truth, it is *not* meant to be ‘true to Walton’. The common belief account deviates from Walton’s original theory in important respects (see especially Sect. 4). The aim of this paper is not necessarily to argue in favour of the common belief account, but simply to explore its shape and tenability: Do we absolutely *have to* bite the bullet concerning the ducklings’ deaths, or is there a viable way out?

### The Proposal

On the common belief account, conditional principles of generation are only valid (or ‘proper’) if they quantify over props whose existence and nature is common belief between participants of the game of make-believe, e.g., all participants believe that the prop exists, all participants believe that all participants believe that the prop exists, etc. A principle such as *P* is thus ruled out. Rather, Gregory’s proposal to pretend that “stumps are bears” has to be understood as (implicitly) quantifying over stumps whose existence and nature is common belief, rather than simply over *all* stumps (within the garden). In other words, the general formula for conditional principles of generation (i.e., “If *p*, then participants are to imagine that *q*”) is reformulated as “If it is common belief between participants that *p*, then participants are to imagine that *q*”. The principle of generation (and its inverse) that guides the stump-game is thus: 
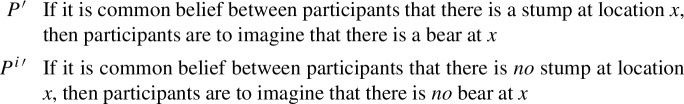
 If the actual world is such that it is common belief between the children that there is a stump left of Gregory (i.e., Gregory believes this, Eric believes this, Gregory believes that Eric believes this, Eric believes that Gregory believes this, etc.^8^), then they are mandated to imagine that there is a bear left of Gregory, i.e., this is true in their game. Similarly, if it is common belief that there is *no* stump at *x*, then it is true in the stump-game that there is *no* bear at *x*. In case the children have no common beliefs about there being a stump at location *x* or not (e.g., because they simply haven’t explored this part of the garden yet), there will be no mandate to imagine that there is a bear, nor a mandate to imagine that there is none. It will thus be *indeterminate* whether there is a bear at *x* in the fiction. As Gregory and Eric collectively explore the garden, their common beliefs about the presence or absence of stumps at different locations will grow in number and hence the set of fictional truths concerning bears will grow, making truth in a game of make-believe a *dynamic* notion.

On this alternative to Walton’s theory, unknown facts about props no longer influence fictional truth. Reconsider the ducklings-game. Even if there actually is a hidden stump at *x*, if the children commonly believe there to be no stump at *x*, there will be a mandate to imagine that there is *no* bear at *x*. Hence it will be true in the ducklings-game that there is *no* bear there. Similarly, in case there is a moss covered boulder at *x* that is commonly mistaken for a stump by Eric and Gregory, there *will* be a mandate to imagine that there is a bear at *x* (contra Walton), and hence a need to paralyse it in the game. Simply put, if the children commonly believe that they have touched all relevant stumps, it will therefore simply be true in their ducklings-game that they rescued the ducklings.

Interestingly, since the account requires *common* belief, it makes truth in a game of make-believe a *collaborative* notion. If Gregory spots the hidden stump at *x* but chooses to ignore it (e.g., because it is getting cold and he wants to quickly finish the game), it will *not* be true in *Eric and Gregory’s game* that there is a bear at *x* (though we might say that this is true in a game that Gregory plays individually with the stumps, see the discussion below). This will even be so in case Eric *also* spots the hidden stump and similarly chooses to ignore it. Gregory may even have noticed that Eric also saw the hidden stump, and hence believe that Eric believes that there is a stump at *x*! Still, if the children don’t acknowledge this ‘publicly’, their beliefs about this stump fall short of *common* belief, and it will *not* be true in the ducklings-game that there is a bear at *x*. Of course, this cannot go on forever. If Gregory and Eric end up face-to-face staring at the stump, or some third party publicly points out the hidden stump to them, then they can no longer avoid the mandate to imagine that there is a bear at *x* (and the need to paralyse it). They would have to change the rules of the game to avoid this fictional truth now.

In the scenario described above, Gregory sees a stump but does not share this information with Eric, thereby causing a discrepancy between the boys’ beliefs about props. Indeed, we can even think of versions of the ducklings-game where such discrepancies are expected and agreed upon. Suppose the garden is quite large and Eric and Gregory agree to split up and each tackle half of the garden. While they explore the garden and touch stumps at various locations, they are mostly out of each other’s sight. Later, they only report to each other *how many* bears they paralysed, but not *where* exactly they found them. Or – even more extreme – Eric and Gregory do not even share any information about the number of bears that they found, but simple say “I have paralysed all bears in my part of the garden”, high-five, and end the game.

On Walton’s original account, in these scenarios there is always just *one* game: the shared or ‘collective’ game played by Eric and Gregory. This game contains a stable set of fictional truths about the number and locations of bears in the garden (determined by the actual stumps in the garden and the shared agreement to pretend “that stumps are bears”) that exists independently from what stumps are actually discovered by the children. Some of those fictional truths (e.g., that there is a bear at some specific location *x* in the East-side of the garden) will only be known to one of the participants (e.g., Gregory, who is is charge of the East-side of the garden). Gregory could share his knowledge of this fictional truth with Eric. Then Eric would become better informed about what was already true in the collective game that he is playing.

It is *not* the case that this kind of separated pretend play (or its equivalent in the representational arts, see footnote 10) is somehow deemed ‘faulty’ or ‘impossible’ according to the common belief account proposed in this paper. In fact, the common belief account allows us to capture the same (groupings of) fictional truths as Walton’s original theory. The only difference is that these fictional truths are now analysed as split up over several games, i.e., Eric’s individual game, Gregory’s individual game, and Gregory and Eric’s collective game. For instance, in case Eric and Gregory only share *how many* bears they paralysed so that, e.g., it becomes common belief that there are 14 stumps in the garden, it will only be true *in Eric and Gregory’s collective game* that there are 14 bears in the garden. In the version of the scenario where *no* information about the details of the boys’ individual conquests are shared, only very general things will be true in Eric and Gregory’s shared game (e.g., Eric paralysed bears in the West-side of the garden). Nothing will be fictionally true in their collective game about the location of the bears, however, as long as Eric and Gregory do not share any information about that. However, we *can* maintain that *in Gregory’s individual game*, it is true that there is a bear at some specific location *x* in the East-side of the garden. Gregory is the only participant of his individual game. Hence if he believes that there is a stump at *x*, then this is also common belief between all participants of Gregory’s individual game. Gregory could share his knowledge of (the location of) the stump with Eric. He would thus enrich Eric and Gregory’s collective game by adding a fictional truth to it.

### What About Representational Art?

Before moving on, I want to address a question that people familiar with Walton’s theory may have had in mind: Walton offers us a theory of the representational arts, whereas I have thus far mostly talked about simple games of make-believe. How does the common belief account translate to plays, films, paintings, sculptures, novels, etc.?

In *Mimesis as Make-Believe*, Walton dedicates large parts of his first chapter to the discussion of the stump-game, detailing how (amongst other things) props, principles of generation and fictional truth work in this simple example of a game of make-believe. He spends the rest of his book exploring how these concepts and principles extend to the (oftentimes more complicated) games we play with representational works of arts (e.g., a painting such as *The Starry Night*, a novel such as *The Hobbit*, a play such as *Hamlet*) as props. The current paper roughly follows Walton’s dialectics. I, first and foremost, focus on the analysis of props, principles of generation and fictional truth in the stump- and ducklings-games, and explore the implications of the alternative common belief account in these simple scenarios. Ideally, like Walton’s original theory, this alternative account of fictional truth can also extend to the representational arts in a coherent and plausible way. For reasons of space, I leave discussion of the implications of the account and its conceptual tenability when it is applied to the representational arts to future research.^9^ Still, I will provide some brief remarks here to sketch how I think this account could roughly work.

As discussed in the previous section, on the common belief account, fictional truth becomes a dynamic and collaborative notion. These traits also translate to fictional truth in games of make-believe we play with representational artworks as props. Importantly, note that here we are still talking about truth *in a game of make-believe*, and not (yet) about ‘representational truth’ (see Sect. 4.2.2).

Concerning collaboration: What kind of predictions the common belief account will lead to when it is applied to representational works of art, is going to depend on whether we think that we are playing a solitary game with the artwork by ourselves, or a collective game together with other people (e.g., with the creator/author of the artwork and/or with all other appreciators of the artwork ). Different media may spur different answers to this question. For instance, when someone reads a novel, we are intuitively inclined to say that they are engaging in a solitary game of make-believe. Hence (assuming positive introspection, see footnote 8) whatever the reader believes about the prop will also be common belief between all participants, and hence function as a basis for fictional truth. However, when I am an audience member of a small-scale interactive theatre performance, it is maybe less obvious that I am engaging in a solitary game of make-believe. Arguably, all audience members are participants of the same game, and hence what we commonly believe about what is happening on stage forms the basis of fictional truth in this collective game.^10^

Concerning dynamicity: As in the stump- and ducklings-games, the common belief account predicts that fictional truth of games of make-believe grows as participants’ (common) beliefs about props increase. Just as Gregory and Eric exploring the garden and finding more and more stumps, generates more and more truths about bears and their locations in the stump-game, a reader processing more and more sentences of *The Hobbit* thereby makes more and more things true in the game that they play with this novel (whereas on Walton’s original theory, everything stated in *The Hobbit* is already true in the game that you are playing with that novel from the get-go).

## Possible Objections

On Walton’s original theory, fictional truths and worlds are independent “from cognizers and their experiences”. They are “like reality, [...] ‘out there,’ to be investigated and explored” (Walton, 1990, p.42). The objections discussed in this section hinge on the intuitive appeal of this idea: that fictional truth should be somehow objective and independent of participants’ mental states.

Before discussing these, however, it is important to specify to what extent the proposed account is in fact incompatible with this idea. On the common belief account, fictional truth is guided by our common beliefs about props, rather than directly guided by these props. In this respect, the account makes explicit fictional truth (contra Walton) depend on cognizers’ mental states or “what people think” (Walton, 1990, p.42). However, as in Walton’s original account, fictional truth is still independent of what participants do and do not *imagine*. If it is common belief that there is a stump at location *x*, then participants of the stump-game will be mandated to imagine that there is a bear at *x*, and hence this will be true in their game. This will be true even if participants for some reason fail to, or refuse to, imagine that there is a bear at *x*. In other words, make-believers *can* still ‘misimagine’ on the proposed account, i.e., in case their imagination is not in line with their common beliefs about props.

### Discovering the Previously Hidden Stump

A defendant of Walton may object along the following lines: Sure, there may exist games with principles of generation such as $P'$, but if participants do not agree on this explicitly, then they are simply not playing that kind of game. If Gregory says “all stumps”, then it simply is *all* stumps (and not covertly all ‘common belief-stumps’). The fact that engagers with fiction indeed accept the consequences of their rules (so specified) is evident from the reaction we, according to Walton, may expect from the children in case they realise, while playing their stump-game, that they were mistaken about there being a stump somewhere:

“False alarm. There isn’t a bear there after all,” [...] “We were mistaken in thinking that, in the world of the game, there was a bear there.” [...] They do not say that fictionally there was a bear which evaporated when they approached, nor that it is *no longer* fictional that a bear was there at the earlier time. (Walton, 1990, p.37)

Likewise, in case the children realise that they were mistaken about there *not* being a stump at *x* (e.g., they find the hidden stump during their stump-game, even though they previously believed that there was no stump at *x*), they will say that it was fictional *all along* that there was a bear at *x*. Walton has a ready-made explanation: The prop was at *x* all along and hence made it true in the fiction all along that there was a bear at *x*. On the common belief account, however, fictional truth is dependent on common beliefs about props. Hence before the children find the stump, it is true in the game that there is *no* bear at *x*, but after they find the stump, it is true that there *is* a bear at *x*!

To avoid the counter-intuitive result of bears evaporating or popping into existence in the stump-game, the common belief account will have to somehow explain the intuition that it is true in the fiction that there was a bear at *x* *all along*. We have to allow for retroactively changing fictional truth. This means that we need to allow for the following in the previously hidden stump scenario (I denote the real and fictional timeline respectively as $\textit{t}_{n}$ and $\textit{t}_{n}'$): At $\textit{t}_{1}$ (when the stump has not been found yet) it is true in the fiction at $\textit{t}_{1}'$ that there is no bear at *x*. At $\textit{t}_{2}$ (when the stump has been found), however, it is true in the fiction at $\textit{t}_{2}'$ *and* at $\textit{t}_{1}'$ that there *is* a bear at *x*. Something that was false in the fiction at $\textit{t}_{1}'$ thus later becomes true in the fiction at $\textit{t}_{1}'$.

The common belief account naturally allows for such retroactive changes in fictional truth. To see why, consider how the discussed principles of generation relate to (fictional) timelines. Arguably, principles of generation for stump- and ducklings-games (whether we go for Walton’s original theory, or for the common belief account), have an implicit time variable: 
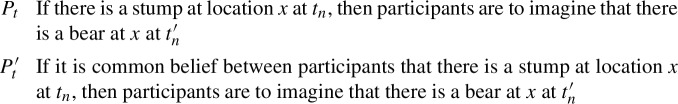
 We can formulate $P_{t}$ and $P_{t}'$ in this way because for the stump- and ducklings-games the fictional timeline parallels the actual timeline, i.e., the fictional events are happening ‘right here, right now’.^11^ Given that stumps usually do not move around, the time variable in $P_{t}$ normally has few consequences for fictional truth. $P_{t}$, for instance, predicts that, in case a stump is relocated (e.g., lifted by an excavator) from location *x* at $t_{1}$ to location *y* at $t_{2}$, it is true in the fiction that a bear moves from *x* at $t_{1}'$ to *y* at $t_{2}'$. For the common belief account, the time variable in $P_{t}'$ has more direct consequences. Reconsider the hidden stump scenario: Once the children find the stump at $t_{2}$, it very probably becomes common belief that there was a stump at *x* *all along*. In other words, at $t_{2}$ the children commonly believe that there was a stump at *x* at $\textit{t}_{2}$ *and* that it must have been there earlier as well, at $\textit{t}_{1}$. Hence, given $P_{t}'$, it becomes retroactively true in the stump-game that there was a bear at *x* at $\textit{t}_{2}'$ *and* $\textit{t}_{1}'$. It is thus *not* true that in the fiction a bear pops into existence at $\textit{t}_{2}'$. Rather, it is true in the fiction (but only from $\textit{t}_{2}$ onwards) that there was a bear there *all along*.

Note that (from $t_{2}$ onwards) it is only true *in the fiction* that there was a bear there all along. Here it is important to distinguish between perspectives ‘internal’ and perspectives ‘external’ to the fiction, and between statements made (by participants or by onlookers) from a perspective that is internal to the fiction, and those made from a perspective that is external to the fiction (or, more precisely, between ‘fictional’ and ‘parafictional’ statements, see e.g., Recanati (2018)). Roughly, are the children continuing their game/pretence when they make their statements (i.e., engaging in internal/fictional discourse), or are they merely reporting on what is true in the fiction (i.e., engaging in external/parafictional discourse)? I suggest that as long as the children adopt an internal perspective, at $t_{2}$ (when they have found the hidden stump), they will *not* (be licensed to) say things like (7) or (8): 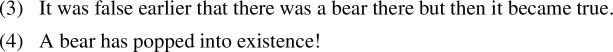 Given $P_{t}'$, these things are simply not true *in the fiction*, and hence should not be said by participants that are playing the stump-game. However, from an external perspective, participants (and onlookers) *are*, of course, licensed to report on retroactive changes in fictional truth. This has to be done in the right way, however. Roughly, in the hidden stump scenario, their natural language utterance should be interpreted as being equivalent to (or entailing) something like (where *p* is the proposition that there is a bear at location *x*) ‘At $t_{1}$ it is the case that in the fiction, it is false that *p* at $\textit{t}_{1}'$; but at $t_{2}$ it is the case that in the fiction, it is true that *p* at $\textit{t}_{1}'$’.^12^ For example, Eric’s father would (be licensed to) say something like (9): 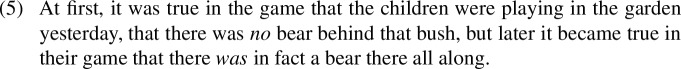 Unless explicitly marked by a fiction-operator, it will not always be easy to distinguish (external) talk *about* the fiction from (internal) continuations of the fictional discourse. This opaqueness may, on the one hand, cloud our judgements concerning felicity of this discourse, and, on the other hand, make it tricky to decide what the account predicts about different example discourses. However, I do note that, when explicitly marked as external through the use of a fiction-operator, statements that report on retroactive changes in fictional truth (e.g., (9)) sound decidedly less problematic or unnatural than internal statements (7) and (8). Still, intuitions may vary here as well, of course. Those who consider the felicity (in the previously hidden stump scenario) of external statements such as (9) an unwelcome prediction, may regard this as a bullet that the defendant of the common belief account has to bite.

Note also that we have independent reasons to allow for retroactively changing fictional truth as (our knowledge of) props develop(s). Introduction of a new character during an act of improvisational storytelling or pretend play can make it retroactively true in the game of make-believe that this person existed all along. This will be true in case their existence was *not* previously contradicted (cf. a scenario in which the children are still exploring the garden), and in case their existence *was* previously contradicted (cf. the hidden stump scenario). For instance, introduction of an adult character half-way through an improvisational theatre performance will (normally) make it retroactively true that this person existed all along (also on Walton’s original account). Likewise, an unreliable campfire storyteller may initially describe their protagonist as an only child and only half-way through their story reveal that they are actually not an only child by introducing a secret sibling.^13^

### Missing Out on Fictional Truths

Suppose twelve children play the ducklings-game and only Gregory failed to see the hidden stump. Or suppose that Mia reads a copy of *The Hobbit* that has pages stuck together and accidentally and unknowingly misses the part where it is stated (amongst other things) that Fíli and Kíli are brothers (*p*). Walton has a ready-made explanation for why it seems that Gregory and Mia miss out on the fictional truths (obvious to others) that there is a bear at *x* and that *p*: They are not perceiving the (part of the) prop that makes this fictional. On the common belief account, however, if one participant doesn’t share a particular belief about a prop, it will not be a *common* belief and hence cannot influence fictional truth. How do we explain our intuition that Gregory and Mia *are *(unlike the others) missing out on fictional truths? I consider two possible strategies that work to different degrees for Gregory’s and Mia’s cases.

#### The Majority’s Truth

First, as Lewis (1978) and Walton (1990) do in their discussion of imported fictional truths, we may switch to a version of the theory that requires mere ‘general’ common belief, i.e., *p* is general common belief in *c* iff *most* members of *c* believe that *p*, *most* members of *c* believe that *most* members of *c* believe that *p*, etc.^14^ Such a move will not affect the discussion of Gregory and Eric’s ducklings-game. They are only two and hence ‘most’ participants is ‘all’ participants. However, in the ducklings-game played by 12 children, it will be general common belief that there is a stump at *x* and hence it will be true in their collective game that there is a bear at *x*. Gregory is thus missing out on a fictional truth of the game that he is playing.

Similarly, because most readers of *The Hobbit* will not have pages stuck together (and believe this about other readers, etc.), *p* *is* true in their collective game and hence Mia is missing out on fictional truth. Note that this strategy only works for Mia if we (contra Walton, and maybe also contra our intuitions) assume that readers of a novel play a collective game, rather than that they all play their own individual game with the prop. Moreover, on this strategy it will *not* be fictional that *p* if the majority of the readers of *The Hobbit* have these two pages stuck together due, for instance, to a large-scale printing error.^15^

#### Representational Truth

The second possible strategy, based on Walton’s notion of fictional truth of a ‘representation’, is probably better suited to Mia’s case. Walton draws a distinction between ‘ad hoc props’ that are pressed into service for a single game of make-believe (e.g., the stumps in the stump-game), and ‘representations’, i.e., things that possess the social function of serving as props in games of make-believe. Artworks such as novels, paintings, sculptures, theatre plays, etc. were specifically designed for this purpose and are thus representations (or works of fiction). Although “[p]eople can play any sort of game they wish with a given work” (Walton, 1990, p.59), only games that accord with a representation’s function are ‘authorised’ for it. For instance, it is *The Hobbit*’s function to serve as a prop in games in which it is true that Fíli and Kíli are brothers, not in games in which they are father and son, or games in which it is undetermined whether they are brothers. To play the latter kind of game is to misuse the work of fiction. Fictional truths of a representation are those propositions that are true in *all* authorised games for this representation.

Simply put, although Mia was (supposedly) using the correct principles of generation, her game was based on only part of the text of *The Hobbit* and hence falls short of an authorised game.^16^ Since fictional truth of a representation is what is true in *all* authorised games, we cannot allow incomplete games to count as authorised. A game based on just the first three sentences of *The Hobbit* could then also count as authorised, and hence hardly anything would be true in *The Hobbit*. Authorised games have to be games that are based on (beliefs about) the entire prop.^17^ Mia, playing an unauthorised game, thus misses out on fictional truths *of the representation* that she is engaging with. An interesting feature of this second strategy is that it only works for representations: Gregory does not miss out on fictional truths *of a representation* since the stumps (being ad hoc props) do not authorise any games.

## Conclusions and Further Questions

In this paper I have explored the shape and tenability of an alternative to Walton’s theory of make-believe (the common belief account) in which unknown facts about props *cannot* influence fictional truth since conditional principles of generation are only valid if they quantify over props whose existence and nature is common belief between participants of the game.

The fact that the common belief account makes fictional truth depend on common beliefs about props raises some additional questions relevant for future research (and possibly also for existing discussions on *imported* fictional truth).

First, one might wonder what happens in cases of graded beliefs about props (see e.g., Stinchcombe (1988); Monderer and Samet (1989) for discussions of graded *common* belief). What if Gregory and Eric’s common belief about there being a stump at some location *x* has a subjective probability of 0.7? It is unclear whether the possibility of graded (common) belief should also lead us to posit the possibility of graded fictional truth. Is it, in this scenario, then also only 0.7 fictionally true that there is bear at *x*? *Within* the pretence at least, this doesn’t seem to make any sense. Surely, Eric would not (be licensed to) say “It is 70% true that there is a bear behind the bush”. Or would Eric’s father (be licensed to) say “The children are not yet fully convinced that there is a stump behind the bush, so it is also not yet entirely true in their game that there is a bear there”? Though the external discourse is less striking, it still sounds odd. Note that the widely adopted Mutual Belief Principle and Analysis 2 (see Sect. 2), which make *imported* fictional truth dependent on common beliefs, raise similar questions. For instance, if the community of origin of some fiction has a graded common belief with a subjective probability of 0.9 that there is no life on other planets, is it then also only 0.9 fictionally true that there is no life on other planets? One way of avoiding these issues is to assume an arbitrary threshold for fictional truth that is influenced by graded (common) belief (e.g., (common) beliefs with a subjective probability of 0.8 or higher influence fictional truth as full (common) belief, (common) beliefs with lower subjective probability do not influence fictional truth), or to simply abstract away from graded belief in the analysis of fictional truth. Another solution might be to somehow limit the graduality of fictional truth to the external perspective. I leave full exploration of the issue of graded (common) belief and its interplay with fictional truth to future research.

Second, on the common belief account, fictional truth is a collaborative notion. But surely, people can also lie about (their beliefs about) props. Can games of make-believe also be guided by mere ‘common acceptance’ (see e.g., Stalnaker (2002)), rather than common belief?

Last, it would be interesting to explore a normative version of the theory in which fictional truth depends on what the children can reasonably be expected to believe about the stumps.

## Footnotes

1 Interestingly, in later work Walton (2013; 2015) distanced himself from this bi-conditional, i.e., he discusses a variety of counterexamples of fictions for which there is a prescription to imagine *p* but it is *not* true in the fiction that *p* (though, see Woodward (2016) on how one can accommodate some of these counterexamples in Walton’s framework). These examples include fictions in fictions, fictions involving plot twists, and illusionary photographs. The hidden stump scenario central to this paper (see the discussion below) is not (reminiscent of) one of the problem cases described by Walton. Importantly, it seems that even on Walton’s later version of his theory, the hidden stump will still make it true that there is a bear behind the bush (and there was no prescription to imagine otherwise). Either way, the discussion of this paper focuses on Walton’s original (1990) theory (though, see footnote 13).

2 Actually, Walton (1990, p.143) emphasises that his ‘primary’/‘secondary’ distinction is not exactly the same as the often used ‘explicit’/‘implicit’ distinction. Unreliable narrators, for instance, may explicitly state *p* while *p* is not a primary truth of the fiction. I gloss over this issue in the current paper.

3 Note that Lewis and Walton offer analyses of what is true *in* a fiction, and not of what is true *according to* some fiction. Arguably, it is not true that *according to* the Sherlock Holmes stories, water is $\hbox {H}_{2}$O (see e.g., Semeijn (2023)).

4 Similarly, for Walton, worlds where all primary truths of *La Grande Jatte* (e.g., that there is a couple strolling in front of a lake) are true *and* water is $\hbox {H}_{2}$O are more similar to the actual world than worlds where all primary truths of *La Grande Jatte* are true and water is *not* $\hbox {H}_{2}$O.

5 See Sect. 4.2 for the notion of *general* common belief. See Vanderschraaf and Sillari (2014) for a discussion of potential issues with the notion of common belief and related concepts.

6 For instance, on Analysis 1, 2, RP and MBP, if the fiction contains a contradiction, everything will be true in it. See Lewis (1983, p.274-275, p.277-278); Walton (1990, p.147-149); Priest (1997); Badura and Berto (2019). Also, as both Lewis (p.273-274) and Walton (p.161-169) acknowledge, there are fictional truths that are neither primary fictional truths, nor are generated by Analysis 1, 2, RP or MBP (e.g., those generated by genre conventions).

7 Nonetheless, for Walton, RP plays a more fundamental role in our interpretative practice, i.e., RP is the rule we *prima facie* have in mind when engaging with fiction, and whenever we do consciously use MBP, we imagine being a member of the relevant artist’s society that uses RP.

8 I assume the principle of positive introspection (see e.g., Rendsvig et al. (2024)): If *a* believes that *p*, then *a* also believes that *a* believes that *p*, etc. In other words, our attitudes are transparent to ourselves. Hence everything that *a* believes will also be ‘common belief’ in a community that only has *a* as a member. The proposal thus does not exclude solitary pretend play as a type of fiction.

9 I thank an anonymous reviewer for suggesting that a potential complication is that for representational arts, issues concerning the *interpretation* (of props) become relevant, and interpretations by (groups of) audience members can be influenced by their (common) beliefs.

10 As in Gregory and Eric’s game, in case there are (parts of) props that are only visible to some participants (e.g., an experimental theatre performance where only half of the audience is able to see what happens behind a screen on stage), we can talk of several games that are played simultaneously by different (groups of) people, containing different sets of fictional truths.

11 This is not necessary for fiction, or even for pretend play, in general (e.g., children can pretend to be roman soldiers under Caesar’s reign or space pirates in a distant future).

12 And not something like ‘It is the case that in the fiction, it is false that *p* at $\textit{t}_{1}'$, but it is true that *p* at $\textit{t}_{2}'$’. There is simply no $t_{n}$ at which this is true. Likewise, a report such as “In Gregory and Eric’s game, a bear has popped into existence!” would be false.

13 Here I am talking about *improvisational* pretend play and storytelling because in cases of plot twists in finished works of fiction (e.g., Christie’s *The Murder of Roger Ackroyd* in which we find out at the end of the story that the narrator, dr. Sheppard, was the murderer (all along) even though it was implied that he was not), we might argue that it was true all along in the game we play with the fiction that there was a secret sibling (or that dr. Sheppard was the murderer). Such fictions (amongst others) made Walton (2013; 2015) qualify his bi-conditional (see footnote 1) (e.g., we are prescribed to (at first) imagine that dr. Sheppard is *not* the murderer, but this is not true in the fiction). Alternatively, you may argue that we were never prescribed to imagine the ‘twisted’ content (e.g., that dr. Sheppard is not the murderer) in an objective sense, and hence this was never fictional (see e.g., Woodward (2016)).

14 ‘Most’ can be understood as ‘more than 50%’ or we might work with a higher threshold. Alternatively, we can allow for a graded (or ‘fuzzy’) notion of fictional truth. See also the discussion in Sect. 5.

15 Easter eggs in films and videogames arguably have a similar effect.

16 This is true independently of whether the majority of readers plays such a game.

17 This makes representational truth, unlike truth in a game of make-believe, a non-dynamic notion (e.g., what is true in *The Hobbit* does not change over time).
